# *PIK3CA* Mutations and Co-Mutations in Operated Non-Small Cell Lung Carcinoma

**DOI:** 10.3390/jcm13237472

**Published:** 2024-12-08

**Authors:** Salih Cokpinar, Ibrahim Halil Erdogdu, Seda Orenay-Boyacioglu, Olcay Boyacioglu, Nesibe Kahraman-Cetin, Ibrahim Meteoglu

**Affiliations:** 1 Department of Thoracic Surgery, School of Medicine, Aydin Adnan Menderes University, Aydin 09010, Türkiye; salih.cokpinar@adu.edu.tr; 2 Department of Molecular Pathology, School of Medicine, Aydin Adnan Menderes University, Aydin 09010, Türkiye; ibrahim.halil.erdogdu@adu.edu.tr (I.H.E.); nesibe.cetin@adu.edu.tr (N.K.-C.); imeteoglu@adu.edu.tr (I.M.); 3 Department of Medical Genetics, School of Medicine, Aydin Adnan Menderes University, Aydin 09010, Türkiye; 4 Faculty of Engineering, Aydin Adnan Menderes University, Aydin 09010, Türkiye; oboyaci@adu.edu.tr

**Keywords:** NSCLC, *PIK3CA* mutation, co-mutation, smoking history

## Abstract

**Background:** Understanding *PIK3CA* mutations and co-mutations in non-small cell lung carcinoma (NSCLC) is critical to developing personalized treatment strategies. Therefore, this study aims to investigate *PIK3CA* mutations and the accompanying somatic variations in NSCLC. **Methods:** This retrospective study included 98 patients over 18 years of age who were diagnosed with NSCLC, operated on, and referred to the Molecular Pathology Laboratory between January 2019 and June 2024 for next-generation sequencing panel tests and *ALK-ROS1* FISH analysis. **Results:** All patients were found to carry *PIK3CA* mutations. Among the 98 NSCLC patients analyzed, 16 (16.33%) were female and 82 (83.67%) were male. The average age of the patients was 64.53 ± 9.63 years, with an age range of 38–84 years, and the majority were 50 years or older. Of the cases, 51 presented the adenocarcinoma subtype, while the remaining 47 showed the squamous cell carcinoma subtype. A smoking history was present in 77 (78.57%) patients, while 21 (21.43%) had no smoking history. The most frequently detected pathogenic or likely pathogenic *PIK3CA* variations were *c.1633G>A* p.E545K (32.65%), *c.1624G>A* p.E542K (11.22%), *c.3140A>G* p.H1047R (11.22%), *c.3140A>T* p.H1047L (5.10%), *c.1357G>C* p.E453Q (4.08%), and *c.3143A>G* p.H1048R (2.04%). The top 10 mutations that most commonly accompanied *PIK3CA* variations were *KRAS*, *NF1*, *TP53*, *EGFR*, *PTEN*, *BRAF*, *KIT*, *CDKN2A*, *SMARCA4*, and *ATM* mutations, respectively. **Conclusions:** *PIK3CA* variations, along with other gene variations, may influence cancer progression and thus may play a crucial role in the determination of targeted treatment strategies.

## 1. Introduction

Non-small cell lung cancer (NSCLC) constitutes approximately 85% of lung cancers and is characterized by genetic heterogeneity. Within this heterogeneity, mutations in the phosphatidylinositol-3-kinase (PI3K) pathway, particularly mutations in the phosphatidylinositol-4,5-bisphosphate 3-kinase catalytic subunit alpha (*PIK3CA*) gene, play a significant role. *PIK3CA* mutations contribute to cancer development by affecting cellular growth and proliferation processes [1,2].

The *PIK3CA* gene encodes the catalytic subunit of the PI3K enzyme complex. The PI3K pathway is responsible for regulating cellular growth, proliferation, and survival processes by receiving signals from receptors on the cell surface [3]. Mutations in the *PIK3CA* gene lead to the constitutive activation of this pathway, thereby disrupting cellular control mechanisms [4]. In NSCLC, *PIK3CA* mutations are commonly concentrated in the exon 9 (E545K) and exon 20 (H1047R) regions [5,6].

In NSCLC, *PIK3CA* mutations are frequently observed alongside mutations in other genes [7]. These co-mutations play a critical role in determining the tumor’s biology and its response to therapy. Notable co-mutations include *EGFR, KRAS, TP53*, and *ALK* rearrangements [8,9]. The presence of these mutations alongside *PIK3CA* mutations in NSCLC can lead to more aggressive tumor behavior and increased resistance to treatment [10,11,12].

Patients with *PIK3CA* mutations may respond to therapies that target the PI3K pathway [13]. These therapies include the use of PI3K inhibitors and mTOR inhibitors. Additionally, the coexistence of *PIK3CA* mutations with other mutations, such as *EGFR* or *ALK*, suggests that combination therapies might be effective [10].

Understanding *PIK3CA* mutations in NSCLC is crucial for developing personalized treatment strategies. Furthermore, since these mutations can coexist with other genetic variations, identifying co-mutations is also a significant factor in determining treatment strategies for NSCLC [14]. While there are a limited number of studies on this topic in the literature, no studies from Türkiye have been reported. Therefore, this retrospective study aims to investigate *PIK3CA* mutations in NSCLC and the accompanying somatic variations associated with these mutations.

## 2. Materials and Methods

### 2.1. Ethics Approval

This study received approval from the institutional non-interventional clinical research ethics committee of the School of Medicine in Aydin Adnan Menderes University (2024/139). All specimens were collected in compliance with institutional review board-approved protocols and anonymized to maintain patient confidentiality. The principles of the Declaration of Helsinki were adhered to. Individual consent for this retrospective analysis was not obtained as per the ethics committee decision.

### 2.2. Patients

In this retrospective study, a total of 220 files of patient aged 18 and over who were tested in the Molecular Pathology Laboratory of Aydin Adnan Menderes University School of Medicine Hospital between January 2019 and June 2024 were screened. Among them, 98 cases were included, all of which had been diagnosed with NSCLC, had undergone surgery, had next-generation sequencing (NGS) panel tests, had *ALK-ROS1* FISH analysis performed, and were found to have *PIK3CA* mutations. For the included cases, age at diagnosis, gender, diagnostic subgroup, and smoking history were recorded. Patients with NSCLC who were not operated on, or who did not undergo NGS panel tests and FISH analysis, were excluded from this study.

### 2.3. Lung Cancer NGS Panel Analysis

Surgical resection specimens were collected from the patients for routine treatment purposes. The specimens were fixed in formalin, embedded in paraffin blocks, and stored at room temperature. Sections in 10 µm thickness were deparaffinized and placed in a tube containing proteinase K and lysis buffer at 56 °C for 12–24 h. Following lysis, DNA was extracted using a commercial DNA isolation kit (QIAamp DNA FFPE Tissue Kit, Qiagen, Hilden, Germany). Binding, washing, and elution steps were carried out according to the manufacturer’s instructions. The DNA purity and concentration were assessed using a NanoDrop spectrophotometer and a Qubit fluorometer (Thermo Fisher Sci., Waltham, MA, USA).

In the NGS library preparation step, the isolated DNA samples were subjected to fragmentation using an enzymatic method. Adapter ligation was performed on the fragmented DNA using a library preparation kit (NEBNext Ultra II DNA Library Prep Kit, New England Biolab, Ipswich, MA, USA). After ligation, library fragments were amplified by PCR, enriching the target region. The size and quantity of the prepared libraries were assessed using an Agilent 2100 Bioanalyzer and a Qubit fluorometer. The libraries were sequenced to detect the *PIK3CA* mutations and co-mutations on the Illumina MiniSEQ NGS platform (MiniSEQ, MN00676, Illumina, Hayward, CA, USA) according to the manufacturer’s instructions and using the Lung Cancer NGS panel (DHS-005Z-12 and DHS-005Z-96, Qiagen) [15]. The QIAseq Targeted DNA Panels for lung cancer include complete exonic regions of 72 genes and 10 base pairs spanning intron-exon junctions.

Raw data were obtained in FASTQ format and subjected to quality control using FASTQC software v.1.0.0. The cleaned sequences were aligned to the reference human genome (GRCh38) using the Burrows-Wheeler Aligner (BWA) software v.1.1.5. Data analysis was performed with MiniSEQ software v.2.3, and quality indicators were evaluated using QCI analysis (Qiagen). Variant selection was conducted using Clinical Insight and Ingenuity software v.8.1.202021 (Qiagen). The data were visually inspected through IGV 2.8.2 software. As a result of these analyses, the identified variants were classified as pathogenic, likely pathogenic, variants of uncertain significance, likely benign, or benign. Additionally, variants were categorized according to clinical significance and target drug values based on the guidelines from the American Society of Clinical Oncology, Association for Molecular Pathology, College of American Pathologists, and American College of Medical Genetics and Genomics.

### 2.4. ALK/ROS1 Rearrangement FISH Analysis

Fluorescence in situ hybridization (FISH) was performed using ALK IQFISH Break-Apart Probe and ROS1 IQFISH Break-Apart Probe (Dako Omnis, Agilent, Santa Clara, CA, USA) dual-color probe kits. Sections of 4 µm thickness from the paraffin blocks were placed on positively charged slides and deparaffinized. The sections were then dehydrated in 70%, 80%, and 100% alcohol, and treated with sodium thiocyanate and protease solution. After dehydration in alcohol, the sections were air-dried. The probe kits were denatured at 80 °C for 5 min and applied to the sections in a humid environment for 12 h. Following the hybridization, the sections were washed with sodium saline citrate at room temperature. Preparations were then air-dried and stained with DAPI away from light. Signals for *ALK* and *ROS1* in at least 50 cells were visualized using a fluorescence microscope (Olympus BX51) with 1000× magnification and immersion oil, and utilizing DAPI, FITC, and TRITC dual and triple filters. *ALK/ROS1* rearrangement was considered positive if atypical staining was observed in ≥15% of tumor cells.

### 2.5. Statistical Analysis

The SPSS (Statistical Package for Social Sciences for Windows v.22.0, SPSS Inc., Chicago, IL, USA) software was used for statistical analysis of the data. Descriptive statistics were calculated as mean ± standard deviation and percentages.

## 3. Results

Of the 98 cases included in our study, 16 (16.33%) were female and 82 (83.67%) were male NSCLC patients. The mean age and the standard deviation of the patients were 64.53 ± 9.63 years with an age range of 38–84 years, and the majority were aged 50 years and older. Among the studied cases, 51 (80.39% male, 19.61% female) exhibited the adenocarcinoma subtype, while the remaining 47 cases (87.23% male, 12.77% female) showed the squamous cell carcinoma subtype. A history of smoking was present in 77 (78.57%) of the patients, while 21 (21.43%) had no history of smoking. The smoking rate was 82.35% for the adenocarcinoma subtype and 89.36% for the squamous cell carcinoma subtype. Sociodemographic and clinical data for the cases are presented in Table 1.

Across all patients who underwent NGS panel testing, 172 pathogenic and 72 likely pathogenic variations were identified. The top 10 genes with the highest numbers of variations were as follows: *PIK3CA* with 107 variations, *KRAS* with 29 variations, *NF1* with 29 variations, *TP53* with 26 variations, *EGFR* with 14 variations, *PTEN* with 12 variations, *BRAF* with 8 variations, *KIT* with 4 variations, *CDKN2A* with 3 variations, *SMARCA4* with 2 variations, and *ATM* with 1 variation.

The most frequently identified pathogenic and likely pathogenic *PIK3CA* variations in the patients were *c.1633G>A* p.E545K (32.65%), *c.1624G>A* p.E542K (11.22%), *c.3140A>G* p.H1047R (10.20%), *c.3140A>T* p.H1047L (5.10%), *c.1357G>C* p.E453Q (4.08%), and *c.3143A>G* p.H1048R (2.04%). A list of the most frequently observed mutations in the NSCLC patients is presented in Table 2.

**Figure 1:**
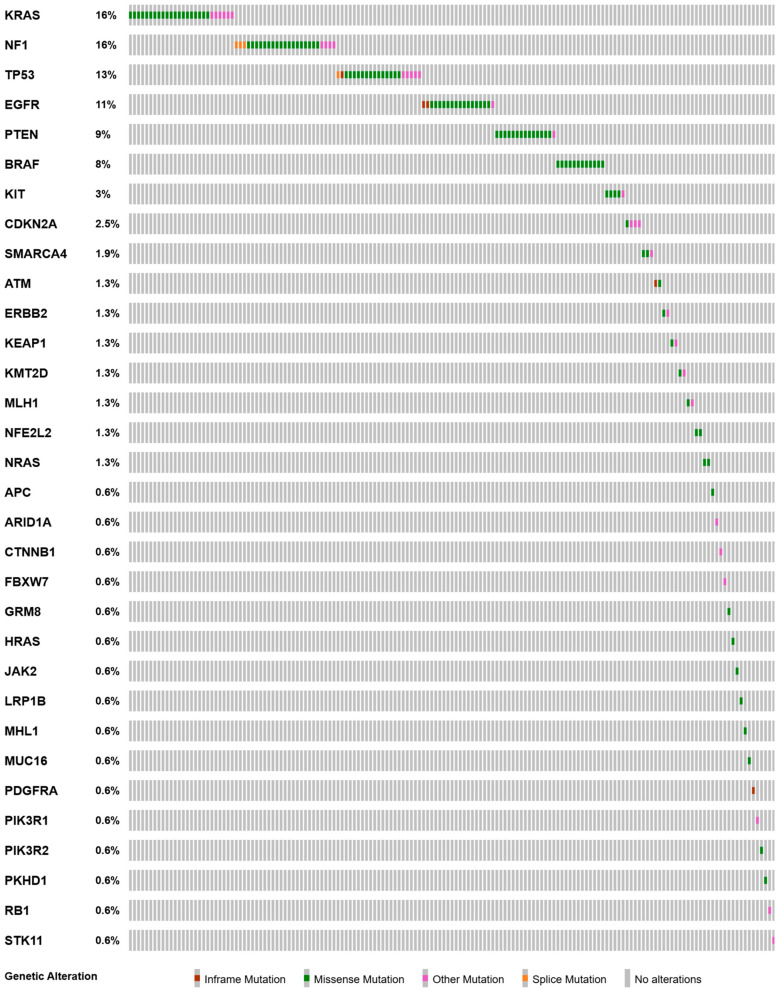
Oncoprint diagram of all mutations co-occuring with *PIK3CA* variants.

The *PIK3CA c.1633G>A* p.E545K pathogenic variant was the variant that was most frequently observed to show co-mutation with other mutations, with a total of 51 co-mutations. This was followed by *PIK3CA c.1624G>A* p.E542K (14 co-mutations) and *PIK3CA c.1637A>G* p.Q546R (8 co-mutations). All of the mutations that co-occuried with *PIK3CA* variants were provided in an oncoprint diagram in Figure 1. The 10 most prevalent co-mutations were *KRAS*, *NF1*, *TP53*, *EGFR*, *PTEN*, *BRAF*, *KIT*, *CDKN2A*, *SMARCA4*, and *ATM* mutations. Detailed information about all gene mutations that co-occurred with *PIK3CA* variants is provided in Supplementary Table S1.

*ALK* rearrangement accompanying *PIK3CA* mutation was detected in five patients using FISH analysis, with a positive detection rate of 5.10%. Similarly, *ROS1* rearrangement accompanying *PIK3CA* mutation was observed in one patient, with a positive detection rate of 1.02%. All *ALK*- and *ROS1*-positive cases were found in adenocarcinoma patients, and no *ALK* or *ROS1* fusions were detected in patients with squamous cell carcinoma.

**Figure 2:**
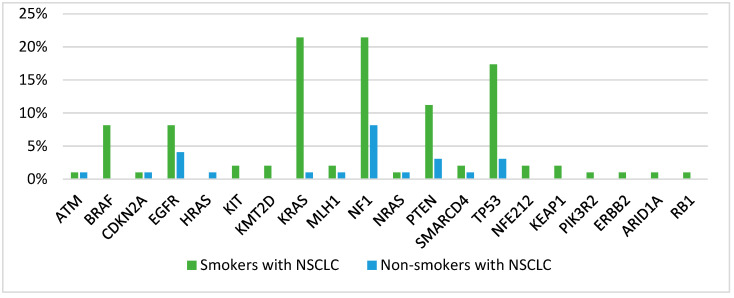
Distribution of *PIK3CA* co-mutations based on smoking status in NSCLC patients.

The 10 most common co-mutations among patients who smoked were *KRAS* (21.42%), *NF1* (21.42%), *TP53* (17.35%), *PTEN* (11.22%), *BRAF* (8.16%), *EGFR* (8.16%), *SMARCA4* (2.04%), *KIT* (2.04%), *KMT2D* (2.04%), and *MLH1* (2.04%) mutations. In contrast, among non-smoking patients, the number of co-mutations accompanying *PIK3CA* mutations decreased, with the identified mutations being *NF1* (8.16%), *EGFR* (4.08%), *PTEN* (3.06%), *TP53* (3.06%), *HRAS* (2.04%), *NRAS* (2.04%), *MLH1* (2.04%), *CDKN2A* (2.04%), *ATM* (2.04%), and *KRAS* (1.02%). A more detailed presentation of *PIK3CA* co-mutations in the NSCLC patients based on their smoking status is provided in Figure 2.

When comparing the *ALK* and *ROS1* rearrangements associated with *PIK3CA* mutations, it was found that *ALK* and *ROS1* rearrangements were not observed in non-smoker patients.

## 4. Discussion

The majority of the NSCLC patients in this study were male (83.67%), which is consistent with the literature suggesting that NSCLC is more commonly observed in males. A study by Siegel et al. (2022) reported that lung cancer has a higher incidence in men, who also exhibit a higher mortality rate compared to women [16]. Additionally, the mean age of 64.53 ± 9.63 years found in our study indicates that NSCLC is typically diagnosed at an advanced age. This is supported by a study conducted by Huang et al. (2022), which noted that the incidence of lung cancer increases with age, with the highest incidence occurring in individuals aged 65 years and older [17].

In our study, 78.57% of patients had a history of smoking, which is one of the most significant risk factors for the development of lung cancer [16,18]. A study conducted by Sharma et al. (2022) demonstrated that smoking significantly increases the risk of lung cancer, with there being a much higher incidence rate in smokers compared to non-smokers [18].

Cigarette smoke contains numerous carcinogenic substances which can lead to DNA damage in lung cells, resulting in mutations. These mutations may drive uncontrolled cell growth and contribute to cancer development. It is well-established that patients who smoke have a higher burden of genetic mutations due to exposure to these carcinogens. Particularly, mutations in tumor suppressor genes such as *TP53* and oncogenes like *KRAS* are more frequently observed in individuals who smoke [19]. Similarly, in our study, it was observed that variations in *PIK3CA, TP53*, and *KRAS* were more common among patients who smoked. Additionally, these patients were found to have a higher mutation load. This finding suggests that smoking may increase the accumulation of genetic variations, which could have significant implications for cancer progression. For instance, *TP53* mutations are more frequently observed in smokers, reflecting the harmful effects of smoking on DNA [20]. Additionally, *KRAS* mutations have also been reported more commonly in individuals who smoke and have been associated with poor prognoses [21]. The presence of these mutations in smokers suggests that cancer may progress more aggressively in smokers and that the response to treatment may be poorer.

*PIK3CA* gene variations play a crucial role in cancer development and are frequently detected across various cancer types [4]. For instance, a study conducted by André et al. (2019) reported a high frequency of *PIK3CA* gene mutations in breast cancers [22]. Similarly, *PIK3CA* mutations have been commonly identified in colon, brain, and other organ cancers [4]. In our study, we similarly observed a high prevalence of *PIK3CA* variations among NSCLC patients. This finding further supports the critical role of the *PIK3CA* gene in the mechanisms leading to cancer.

In our study, the most frequently detected variations in the *PIK3CA* gene were *c.1633G>A* p.E545K (32.65%), *c.1624G>A* p.E542K (11.22%), and *c.3140A>G* p.H1047R (11.22%). These variations have also been frequently reported in the literature, with the p.E545K variation, in particular, standing out as the most common co-mutated variation, among others [3,23]. The results of our study are consistent with these findings.

*PIK3CA* mutations (in exons 9 and 20) are commonly found together with other molecular alterations, such as in the *EGFR*, *KRAS*, *ALK*, and *PTEN* genes [24]. In our study, the mutations that most frequently accompanied *PIK3CA* variations were *NF1, KRAS*, and *TP53* mutations. In a study by Zhang et al. (2024), mutations in *PIK3CA*, observed alongside *EGFR, KRAS*, and *TP53* mutations, were frequently seen in patients with NSCLC [25]. In a study by Liang et al. (2015) that was conducted in the Chinese population, other oncogene mutations found in conjunction with *PIK3CA* mutations in NSCLC patients included *KRAS E2* (11 cases), *KRAS E3* (1 case), *BRAF* (2 cases), *EGFR E18* (4 cases), *EGFR E20* (5 cases), *EGFR E21* (28 cases), and *EGFR E19* deletions (37 cases) [26]. In studies by Chaft et al. (2012) and Scheffler et al. (2015), it was concluded that *PIK3CA* mutations in lung adenocarcinomas typically occur simultaneously with *EGFR, KRAS*, and *ALK* mutations [2,27]. In contrast to these studies, our current work suggests that the frequency of *NF1* mutations associated with *PIK3CA* may depend on the sensitivity of the mutation screening method, ethnic differences, and the specific driver mutations examined in other studies. In this regard, our results highlight the co-occurrence of *PIK3CA-NF1* mutation in NSCLC.

Co-mutations accompanying *PIK3CA* mutations may amplify the impact of *PIK3CA* variations on cancer progression, making them significant from a clinical management perspective. For instance, *TP53* mutations can disrupt tumor suppressor gene function, leading to a more aggressive course of the disease [28]. These findings are consistent with other reports. For example, a study conducted by the Cancer Genome Atlas Research Network (2012) demonstrated that *TP53* mutations are prevalent across various cancer types and are associated with a more aggressive disease course [29]. Similarly, *KRAS* mutations have been frequently reported in several cancer types and have been linked to poor prognoses [21]. The frequent occurrence of *KRAS* and *TP53* mutations alongside *PIK3CA* variations in the current study further highlights the role of these genes in NSCLC.

Adenocarcinoma and squamous cell carcinoma are the two main subtypes of NSCLC, each with distinct molecular profiles and clinical characteristics. Adenocarcinoma is typically more common in non-smokers and women, and it is usually located in the peripheral regions of the lung. Genetic variations that are frequently detected in adenocarcinoma include *EGFR*, *ALK*, and *ROS1* mutations [30]. However, in our study, the adenocarcinoma subtype was more frequently observed in males and smokers, which differs from the typical findings in literature. The most commonly detected variants were *PIK3CA*, *NF1*, *KRAS*, and *EGFR.* This discrepancy may be attributed to ethnic differences (which could stem from various factors such as genetics, the environment, culture, socioeconomic status, and access to healthcare) or tumor heterogeneity. This is because oncogenic driver mutations in NSCLC are often associated with specific clinical and pathological characteristics, such as histological subtypes, gender, ethnicity, age, smoking history, and the status of other common oncogenes [31].

Squamous cell carcinoma is typically more prevalent in smokers and is usually located in the central regions of the lung. Commonly detected genetic alterations in this subtype include *FGFR1* amplifications and *DDR2* mutations [32]. In our study, the squamous cell carcinoma subtype was more frequently observed in males and among smokers, which is consistent with the literature. The most frequently detected variants in this subtype were *PIK3CA*, *NF1*, *KRAS*, *BRAF*, and *EGFR* mutations.

Kinase gene fusions, particularly, are one of the rarer mutation types in lung cancer. For example, *ALK, ROS1*, and *RET* fusion mutations are found in 2–7%, 1–2%, and 1–2% of advanced-stage NSCLC patients, respectively [33,34,35]. *ALK*, *ROS1*, and *RET* rearrangement is typically more common among younger individuals, non-smokers, and light smokers who have been diagnosed with adenocarcinoma [36]. In our study, *ALK* and *ROS1* rearrangements were observed at rates of 5.10% and 1.02%, respectively, which is consistent with the literature. However, these rearrangements were not common in non-smokers. This may be due to the fact that we had more detailed information about the smoking histories of the patients than their current smoking status.

In our study, the *PIK3CA* mutation frequently exhibited co-mutations in NSCLC patients. The frequent co-occurrence of *PIK3CA* variations may be significant in determining targeted treatment strategies for NSCLC. In patients with *PIK3CA* variations, the use of drugs targeting these variations could be considered. For instance, PI3K inhibitors may be effective in these patients and could play a crucial role in the personalization of treatment strategies [37].

Intratumoral heterogeneity (ITH) is observed in most tumors during the investigation of the evolutionary trajectories of different cancer types. In lung cancer, factors that affect ITH may include genetic factors, pulmonary neoplastic microenvironmental factors, or metabolic factors [38]. The common mutations identified in our study are those detected after tumor resection; however, these mutations may not be present in all tumor cells in the same way. This reflects the genetic heterogeneity of tumors.

Additionally, our study observed an increased mutation burden in patients who smoked. Understanding the genetic changes induced by smoking may enable more effective treatment planning for these patients [39].

### Limitations

Although our study provides insights into targeted therapy, correlating specific mutation profiles with actual patient outcomes, such as survival or progression rates, would bridge the gap between molecular findings and clinical application. To build on this research, functional studies could be conducted to examine the cellular effects of *PIK3CA* mutations in NSCLC cells. In particular, assays comparing the proliferation, migration, and apoptosis of mutated cells to that of non-mutated cells could contribute to the findings of this study.

## 5. Conclusions

The findings of this study highlight the prevalence of *PIK3CA* variations and their co-mutation profiles in NSCLC patients, emphasizing the potential impact of these variations on the clinical management of and treatment strategies for NSCLC. *PIK3CA* variations, in conjunction with other genetic mutations, can influence cancer progression and, therefore, may play a crucial role in determining targeted therapeutic approaches to cancer. Additionally, the relationship between smoking and genetic variations may contribute to a better understanding of the molecular pathogenesis of NSCLC. In this context, larger-scale and more comprehensive studies may support the integration of these findings into clinical practice.

## Figures and Tables

**Table 1:** Demographic (A) and clinical (B) characteristics of NSCLC patients.

A			(% or Mean ± St Dev)			
**Gender**	Female		16.33%			
	Male		83.67%			
**Mean age**	General		64.53 ± 9.63			
	Female		59.88 ± 8.62			
	Male		65.44 ± 9.60			
**Smoking status**	Smoking		78.57%			
	No Smoking		21.43%			
**Mean age for**	Smokers		64.99 ± 8.94	Female		59.33 ± 9.63
				Male		65.74 ± 8.64
	Non-Smokers		62.86 ± 11.93	Female		60.57 ± 7.81
				Male		64.00 ± 13.67
**B**						
**Subtypes of NSCLC**	**Female**	**Male**	***p* Value**	**Smoking**	**No Smoking**	***p* Value**
**Adenocarcinoma**	19.61%	80.39%	0.36	82.35%	17.65%	0.34
**Squamous Cell Carcinoma**	12.77%	87.23%		89.36%	10.64%	

**Table 2:** Pathogenic and likely pathogenic *PIK3CA* variants in the studied cases.

Codon	Protein	Exon	Pathogenicity Status	Incidence (%)
*c.1633G>A*	p.E545K	10	Pathogenic	32.65
*c.1624G>A*	p.E542K	10	Likely pathogenic	11.22
*c.3140A>G*	p.H1047R	21	Pathogenic	11.22
*c.3140A>T*	p.H1047L	21	Pathogenic	5.10
*c.1357G>C*	p.E453Q	8	Pathogenic	4.08
*c.3143A>G*	p.H1048R	21	Likely pathogenic	2.04
*c.112C>A*	p.R38S	2	Likely pathogenic	1.02
*c.113G>A*	p.R38H	2	Pathogenic	1.02
*c.241G>C*	p.E81Q	2	Pathogenic	1.02
*c.241G>A*	p.E81K	2	Likely pathogenic	1.02
*c.277C>T*	p.R93W	2	Pathogenic	1.02
*c.320A>T*	p.N107I	2	Likely pathogenic	1.02
*c.329A>G*	p.E110G	2	Likely pathogenic	1.02
*c.331A>G*	p.K111E	2	Pathogenic	1.02
*c.1031T>C*	p.V344A	5	Likely pathogenic	1.02
*c.1054G>T*	p. D352H	5	Likely pathogenic	1.02
*c.1073C>G*	p.T358R	6	Likely pathogenic	1.02
*c.1134T>G*	p.C378W	6	Likely pathogenic	1.02
*c.1136_1137insGA...*	p.N380_P381insIDPN	6	Likely pathogenic	1.02
*c.1173A>G*	p.I391M	7	Pathogenic	1.02
*c.1258T>C*	p.C420R	8	Pathogenic	1.02
*c.1357G>A*	p.E453K	8	Pathogenic	1.02
*c.1384A>G*	p.T462A	8	Likely pathogenic	1.02
*c.1396C>G*	p.P466A	8	Likely pathogenic	1.02
*c.1437G>T*	p.W479C	9	Likely pathogenic	1.02
*c.1451T>C*	p.V484A	9	Likely pathogenic	1.02
*c.1616C>G*	p.P539R	10	Pathogenic	1.02
*c.1624G>C*	p.E542Q	10	Pathogenic	1.02
*c.1624G>A*	p.E542K	10	Pathogenic	1.02
*c.1633G>A*	p.E545K	10	Pathogenic	1.02
*c.1633G>C*	p.E545Q	10	Pathogenic	1.02
*c.1634A>C*	p.E545A	10	Pathogenic	1.02
*c.1637A>G*	p.Q546R	10	Pathogenic	1.02
*c.1638G>C*	p.Q546H	10	Pathogenic	1.02
*c.2761A>C*	p.I921L	19	Likely pathogenic	1.02
*c.2908G>A*	p.E970K	20	Pathogenic	1.02
*c.3019G>A*	p.G1007S	21	Likely pathogenic	1.02
*c.3052G>C*	p.D1018H	21	Likely pathogenic	1.02
*c.3068G>A*	p.R1023Q	21	Likely pathogenic	1.02
*c.3073A>G*	p.T1025A	21	Pathogenic	1.02
*c.3127A>G*	p.M1043V	21	Pathogenic	1.02
*c.3131A>T*	p.N1044I	21	Likely pathogenic	1.02
*c.3143A>G*	p.H1048R	21	Pathogenic	1.02
*c.3145G>C*	p.G1049R	21	Pathogenic	1.02
*c.3155C>A*	p.T1052K	21	Pathogenic	1.02

## Data Availability

Additional data are available upon request.

## Supplementary Materials

The following supporting information can be downloaded at: https://www.mdpi.com/article/10.3390/jcm13237472/s1, Table S1. *PIK3CA* pathogenic and likely pathogenic co-mutations detected in cases.
