# Supplementary material for: PIK3CA Mutations and Co-Mutations in Operated Non-Small Cell Lung Carcinoma
Source: J Clin Med. 2024 Dec 8;13(23):7472. doi: 10.3390/jcm13237472 (PMC11641866; doi:10.3390/jcm13237472)
Supplement: Supplementary file 1 [file jcm-13-07472-s001.zip › jcm-3207376-supplementary.pdf]

Supplementary Table S1. *PIK3CA* pathogenic and likely pathogenic co-mutations detected in cases

Gene	Codon	Protein	Exon	Gene	Codon	Protein
PIK3CA	*c. 1637A>G*	p. Q546R	10	*KRAS*	*c.35G>T*	p.G12V
				NF1	*c.4603G>A*	p.D1535N
				NF1	*c.5450C>A*	p.S1317Y
				NF1	*c.563C>T*	p.S1844F
				PIK3R2	*c.1604_1606delTC*	p.I635fs*2
				ARID1A	*c.5372C>A*	p.S1791*
				ATM	*c.8432dupA*	p.S2812fs*3
				ATM	*c.7865C>T*	p.A2622V
PIK3CA	*c.1633G>A*	p.E545K	10	*BRAF*	*c.1405G>A*	p.G469R
				BRAF	*c.2104G>A*	p.D702N
				BRAF	*c.1799T>A*	p.V600E
				EGFR	*c.2236_2250delGAATTAA*	p.E746_A750del
				EGFR	*c.2235_2249delGGAATTAA*	p.E746_A750del
				HRAS	*c.182A<t*	p.q61l
				KEAP1	*c.1430dupG*	p.F478fs*2
				KEAP1	*c.1708+1G>T*	-
				KIT	*c.1676T>A*	p.V559D
				KRAS	*c.437C>T*	p.A146V
				KRAS	*c.35G>C*	p.G12A
				KRAS	*c.38G>A*	p.G13D
				KRAS	*c.35G>A*	p.G12D
				KRAS	*c.35G>T*	p.G12V
				MLH1	*c.1007G>A*	p.G336D
				MUC16	*c.30539C>G*	p.S10180*
				NF1	*c.1400C>T*	p.T467I

				NF1	*c.4773-1G>T*	
				NF1	*c.2374C>T*	p.L792F
				NF1	*c.1400C>T*	p.T467I
				NF1	*c.2325+3A>G*	-
				NF1	*c.2491C>T*	p.L831L
				NF1	*c.2374C>T*	p.L792F
				NF1	*c.2374C>T*	p.L792F
				NFE2L2	*c.91G>A*	p.G31R
				NRAS	*c.35G>A*	p.G12D
				PKHD1	*c.8797_8797+1delGGinsTT*	-
				PTEN	*c.397G>A*	p.V133I
				PTEN	*c.407G>A*	p.C136Y
				PTEN	*c.407G>A*	p.C136Y
				RB1	*c.19C>T*	p.R7*
				SMARCA4	*c.1120_1121delGA*	p.E374fs*3
				STK11	*c.759C>A*	p.Y253*
				TP53	*c.578A>T*	p.H193L
				TP53	*c.848G>C*	p.R283P
				TP53	*c.548C>G*	p.S183*
				TP53	*c.821T>A*	p.V274D
				TP53	*c.701A>G*	p.Y234C
				TP53	*c.202G>T*	p.E68*
				TP53	*c.743G>A*	p.R248Q
				TP53	*c.546_559+4delCTCAGATAGCGATGGTGA*	-
				TP53	*c.469G>T*	p.V157F
				TP53	*c.1036G>T*	p.E346*
				TP53	*c.298delC*	p.Q100fs*23
				TP53	*c.713G>A*	p.C238Y

PIK3CA	c.1384A>G	p.T462A	8	GRM8	c.697G>T	p.E233*
				PTEN	c.397G>A	p.V133I
				TP53	c.1015G>T	p.E339*
PIK3CA	c.1616C>G	p.P539R	10	NF1	c.2693T>C	p.L898P
				NRAS	c.38G>A	p.G13D
PIK3CA	c.1134T>G	p.C378W	6	KMT2D	c.1080delC	p.Q3601fs*57
				MLH1	c.469G>T	p.Y157fs*3
PIK3CA	c.3073A>G	p.T1025A	21	EGFR	c.2573T>G	p.L858R
				EGFR	c.2281G>T	p.D761Y
PIK3CA	c.2761A>C	p.I921L	19	CDKN2A	c.181G>T	p.E61*
				SMARCA4	c.1993G>T	p.E665*
				TP53	c.565G>T	p.A189S
PIK3CA	c.1396C>G	p.P466A	8	EGFR	c.2281G>A	p.D761N
PIK3CA	c.329A>G	p.E110G	2	BRAF	c.1787G>A	p.G596D
				KRAS	c.36T>C	p.G12G
PIK3CA	c.1746-2A>T		14	CDKN2A	c.238C>T	p.R80*
				PTEN	c.1621A>C	p.V166fs*14
PIK3CA	c.1746-2A>T		14	CDKN2A	c.238C>T	p.R80*
				PTEN	c.1621A>C	p.V166fs*14
PIK3CA	c.3143A >G	p.H1048R	21	EGFR	c.2252_2275delCA...	p.T751_E758delTS...
				PTEN	c.407G>A	p.C136Y
				BRAF	c.1408A>G	p.T470A
				KRAS	c.35G>T	p.G12V
				KRAS	c.15A>C	p.K5N
				EGFR	c.2236_2250delGAATT..	p.A746_A750del
PIK3CA	c.1451T>C	p.V484A	9	APC	c.49396G>A	p.G1466
				FBXW7	c.1177C>T	p.R939*
				NF1	c.1400C>T	p.T467I

				NF1	*c.7553-1G>T*	-
				PIK3R1	*c.643delA*	p.S215fs*9
				PTEN	*c.106G>A*	p.G36R
PIK3CA	*c.3068G>A*	p.R1023Q	21	*BRAF*	*c.1787G>A*	p.G596D
				KRAS	*c.36T>C*	p.G12G
PIK3CA	*c.1031T>C*	p.V344A	5	*EGFR*	*c.2252_2275delCA...*	p.T751_E758delTS…..
				BRAF	*c.1408A>G*	p.T470A
				KRAS	*c.35G>T*	p.G12V
				KRAS	*c.15A>C*	p.K5N
PIK3CA	*c.3145G>C*	p.G1049R	21	*EGFR*	*c.2240_2254delTAAGA*	p.L747_T751del
				NF1	*c.2325+3A>G*	-
				NF1	*c.1400C>T*	p.T467I
				PTEN	*c.407G>A*	p.C136Y
PIK3CA	*c.1357G>C*	p.E453Q	8	*EGFR*	*c.2485G>A*	p.E829K
				KIT	*c.1668G>C*	p.Q556H
				NF1	*c.2373C>T*	p.L792F
PIK3CA	*c.1396C>G*	p.P466A	8	*EGFR*	*c.2281G>A*	p.D761N
PIK3CA	*c.3052G>C*	p.D1018H	21	*EGFR*	*c.2485G>A*	p.E829K
				KIT	*c.1668G>C*	p.Q556H
PIK3CA	*c.1634A>C*	p.E545A	10	*KRAS*	*c.35G>T*	p.G12V
PIK3CA	*c.331A>G*	p.K111E	2	*KRAS*	*c.35G>T*	p.G12V
PIK3CA	*c.3155C>A*	p.T1052K	21	*BRAF*	*c.1799T>A*	p.V600E
PIK3CA	*c.3019G>A*	p.G1007S	21	*KIT*	*c.1676T>A*	p.V559D
				KRAS	*c.35G>C*	p.G12A
				KRAS	*c.38G>A*	p.G13D
PIK3CA	*c.1073C>G*	p.T358R	6	*BRAF*	*c.1331G>A*	p.R444Q
				EGFR	*c.2300C>T*	p.A767V
				EGFR	*c.2585T>A*	p.L862Q

				ERBB2	*c.2524G>T*	p.V642L
				KRAS	*c.39C>T*	p.G13G
PIK3CA	*c.1437G>T*	p.W479C	9	*BRAF*	*c.1331G>A*	p.R444Q
				EGFR	*c.2300C>T*	p.A767V
				EGFR	*c.2585T>A*	p.L862Q
				ERBB2	*c.2524G>T*	p.V642L
				KRAS	*c.39C>T*	p.G13G
PIK3CA	*c.277C>T*	p.R93W	2	*TP53*	*c.380C>T*	p.S137F
PIK3CA	*c.3131A>T*	p.N1044I	21	*NF1*	*c.480-1G>T*	-
				TP53	*c.949dupC*	p.Q219*
				PTEN	*c.655C>T*	p.Q219*
PIK3CA	*c.320A>T*	p.N107I	2	*PTEN*	*c.397G>A*	p.V133I
				PTEN	*c.407G>A*	p.C136Y
				TP53	*c.460_465dupGGACC*	p.G154_T155dup
PIK3CA	*c.329A>G*	p.E110G	2	*BRAF*	*c.1787G>A*	p.G596D
				KRAS	*c.36T>C*	p.G12G
PIK3CA	*c.1054G>T*	p.D352H	5	*KMT2D*	*c.11759T>A*	p.L3920Q
				NF1	*c.2374C>T*	p.L792F
				NF1	*c.4675G>T*	p.E1559*
				PDGFRA	*c.2212_2214delGAT*	p.D738del
				TP53	*c.800G>C*	p.R267P
PIK3CA	*c.16+33G>A*	p.E545K	10	*KRAS*	*c.34G>T*	p.G12C
PIK3CA	*c.1624G>A*	p.C136Y	10	*LRP1B*	*c.214G>T*	p.E72*
				EGFR	*c.2235_2249delGGAATTA*	p.E746_A750del
				CTNNB1	*c.110C>G*	p.S37C
				NF1	*c.1400C>T*	p.T467I
				PTEN	*c.407G>A*	p.C136Y
PIK3CA	*c.3140A>T*	p.H1047L	21	*BRAF*	*c.1396G>A*	p.G466R

				KRAS	*c.35G>A*	p.G12D
				KRAS	*c.35G>T*	p.G12V
				KRAS	*c.34G>T*	p.G12C
				NF1	*c.1400C>T*	p.T467I
PIK3CA	*c.241G>C*	p.E81Q	2	*EGFR*	*c.2485G>A*	p.E829K
				KIT	*c.1668G>C*	p.Q556H
				JAK2	*c.1849G>T*	p.V617F
PIK3CA	*c.2761A>C*	p.I921L	19	*CDKN2A*	*c.181G>T*	p.E61*
				TP53	*c.565G>T*	p.A189S
				SMARCA4	*c.1993G>T*	p.E665*
PIK3CA	*c.1624G>A*	p.E542K	10	*KRAS*	*c.34_35delGGinsTT*	p.G12F
				KRAS	*c.34G>T*	p.G12C
				TP53	*c.1624G>A*	p.P151S
				MHL1	*c.848A>G*	p.Y283C
				KRAS	*c.35G>A*	p.G12D
				TP53	*c.488A>G*	p.Y163C
				NFE2L2	*c.1958G>A*	p.E79K
				NF1	*c.1400C>T*	p.T467I
				NF1	*c.2325+3A>G*	-
				NF1	*c.2374C>T*	p.L792F
				PTEN	*c.407G>A*	p.C136Y
				PTEN	*c.397G>A*	p.V133I
